# Development of Trait Emotional Intelligence in Response to Childbirth: A Longitudinal Couple Perspective

**DOI:** 10.3389/fpsyt.2020.560127

**Published:** 2020-10-27

**Authors:** Sarah Galdiolo, Justine Gaugue, Moïra Mikolajczak, Patty Van Cappellen

**Affiliations:** ^1^Department of Psychology, University of Mons, Mons, Belgium; ^2^Department of Psycholog, Catholic University of Louvain, Louvain-la-Neuve, Belgium; ^3^Social Science Research Institute, Duke University, Durham, NC, United States

**Keywords:** trait emotional intelligence, childbirth, dyadic perspective, developmental trajectories, parents

## Abstract

The aim of the current paper was to investigate the influence of childbirth on parents' trait emotional intelligence (EI). A three-wave longitudinal research program (during the second trimester of pregnancy, at 6 months postpartum, and at 1 year postpartum) using the Actor-Partner Interdependence Model with a hierarchical linear modeling was conducted on 204 parental couples with parental group (i.e., primiparous and multiparous parents) as a time-invariant predictor and the partner's EI development as a time-varying covariate. Results showed that parents' EI was stable, except for Self-Control that increases after childbirth. Moreover, there was a significant negative association between the actor's and the partner's development around childbirth. Childbirth pushes parents to function in dyad rather than individually. Compensatory effects may be observed between both parents in terms of emotional management of parenting: When one partner cannot cope emotionally with parenting, the other partner would compensate and better manage the emotional aspects of parenting. The discussion underlined the importance of the dyadic perspective in understanding the childbirth experience, specifically the parents' receptivity to variation in their partners' emotional levels.

Although we all experience various emotions throughout our lives, we markedly differ in the ways we process these emotions. How people process emotion-related information and react to emotional events may be influenced by their trait emotional intelligence (EI), as conceptualized by Petrides and Furnham (1). People with high trait EI can accurately identify their own emotions as well as those of others. These people are also able to express emotions in socially acceptable manners, understand their causes and consequences, regulate them when they are inappropriate in a given context or incongruent to their goals. Furthermore, they often use emotions to improve their social relationships and inform their thoughts as well as actions. Given the significant influence trait EI has on people's well-being, health, and relationships (2), researchers have been investigating whether trait EI may show any improvement as a result of EI training (Pérez-Gonzaléz et al., in preparation). It appears that trait EI is subject to change, as reflected in Trait EI Questionnaire scores (TEIQue; +12% in self-reports and +6.6% in reports by spouses or friends). Such results further raise the question of whether EI may be changed after significant life events, such as having a child, one that could drastically alter one's life.

The current study was a part of a 3-wave longitudinal research program examining the developmental trajectory of parents' personality traits, attachment orientations, and EI around childbirth (i.e., pregnancy, 6 months old, and 1 year old). First, previous results (3–5) showed that (a) parents' personality traits and attachment orientations did not change after childbirth, except for father's Extraversion, which decreased over time and (b) parental couples followed the same developmental trajectory. Second, our preliminary two-wave study (6) showed that childbirth did not influence parents' EI. However, this preliminary study had some limitations, e.g., only two waves of measurement and considering an individual rather than a dyadic perspective. Thus, the question remains: Does childbirth lead to parents' EI changes? In tackling this question, the present study addresses three broad points. First, the Social Structural Theory [SST; (7)] and the social investment principle [SIP; (8)] could explain why EI may change over time. The SST posits that a change in roles (e.g., parent) prompts subsequent psychological changes to adjust to the role (e.g., better emotion management). The SIP states that the investment in social institutions (e.g., parenthood) is embodied in social roles (e.g., parent), which leads to increasing expectations for the pertinent actors. These expectations may include emotional stability, social responsibility, and prosocial behaviors, which then leads to personal growth. Consequently, having a child would lead to new social roles followed by psychological changes to adjust to these roles, such as developing a better adapted EI profile. Second, childbirth influences parents' life at two levels: at the level of couple and at the level of gender roles. As such, childbirth leads couples to experience shared emotional experiences and problems (e.g., high correlation of postpartum depression between both partners). In addition to the couple's level of parenting experience, a gender gap could also be observed after childbirth: Parents become more traditional in their gender-role attitudes following the birth of a child (9) and women tend to change more than men. Our current study investigates both levels of changes, i.e., between-couples changes (i.e., both partners would show the same developmental trajectory but this trajectory differs from couple to couple) and within-couples changes (i.e., different developmental trajectory between men and women but a similarity between couples). Third, our study also raises the question of the Transition to Parenthood Hypothesis vs. New Baby Hypothesis: Do primiparous and multiparous parents change after childbirth? Katz-Wise et al. (9) showed that psychological changes occurred for both primiparous and multiparous parents over time, but changes were greater for primiparous than multiparous parents.

The first objective of the current study is to test for intra-individual changes in EI around childbirth. Based on the SST and SIP, we hypothesized that adults who have recently had a baby would show higher EI than non-parents. The second objective involves testing the Within- and Between-Couples Changes Hypotheses. The last objective is to test the Transition to Parenthood Hypothesis vs. New Baby Hypothesis.

## Methods

### Sample and Procedure

Longitudinal data were collected from a sample of 204 heterosexual, cohabiting, parental couples (*N* = 143 primiparous, *N* = 60 multiparous, and *N* = 1 combination primiparous—multiparous), corresponding to 408 parents (*N* = 204 mothers and *N* = 204 fathers). The primiparous parents' ages ranged from 18 to 45 years old (*M* = 28.61, *sd* = 4.21 for the overall sample; *M* = 27.47, *sd* = 3.46, and *M* = 29.76, *sd* = 4.58, respectively, for mothers and fathers) and the multiparous parents' ages ranged from 22 to 43 years old (*M* = 31.93, *sd* = 4.07 for the overall sample; *M* = 30.56, *sd* = 3.53 and *M* = 33.26, *sd* = 4.14, for mothers and fathers, respectively). A control group was also recruited, which consisted of 215 cohabiting non-parents (*N* = 125 women and *N* = 90 men) whose ages ranged from 19 to 52 years old (*M* = 26.24, *sd* = 5.62 for the overall sample; *M* = 25.21, *sd* = 4.79 and *M* = 27.73, *sd* = 6.40, for women and men, respectively). On account of differences in gender distribution between the target and control group, gender was controlled for in the analyses.

Participants were recruited with the assistance of gynecologists at hospitals who gave information about the study to their patients verbally and by flyers. These patients were either (future) parents in the second trimester of pregnancy or childless women who went for routine check-ups (the latter were asked to recruit their partners). Data were first collected on parents and purposefully on non-parents in order to match the couples for age. At each wave of data collection, participants completed a questionnaire on the Internet via LimeSurvey.

As part of a longitudinal research program, the study involved three waves of data collection, which took place at three distinct timepoints in parenthood: pregnancy (*M* = 23.67 pregnancy weeks, *sd* = 8.49), 6 months postpartum (*M* = 25.03 weeks postpartum, *sd* = 4.81), and 1 year postpartum (*M* = 12.76 months postpartum, *sd* = 1.66). With regard to the non-parental couples, two waves of data collection took place within a 6-months interval.

## Measures

### Sociodemographic Variables

Sociodemographic variables collected during the first wave of data collection included gender, date of birth, details of primiparity, and number of weeks of pregnancy.

### Longitudinal Variable: Trait Emotional Intelligence

For each wave of data collection, trait EI was assessed by means of the Trait EI Questionnaire (10). This questionnaire consisted of four factors: Well-Being, Self-Control, Emotionality, and Sociability (see Appendix). A 5-point Likert-type scale (1 = *completely disagree* and 5 = *completely agree*) was used. An overall EI score and scores for each subscale were then obtained. In previous research, the TEIQue has shown high Cronbach's alphas (αs, 0.71–0.91) and hence is considered to be highly reliable with high construct, predictive, and convergent/discriminant validity (10). In our sample, αs were >0.90 for the global score and varied between 0.82 and 0.90 for the different subscale scores.

### Exclusion Criteria

A depression scale and a stressful life events measure were administered in order to identify and exclude from the sample postnatally depressed parents and participants who had experienced disruptive life events. Depression [i.e., Beck Depression Inventory Short Form Items, BDI-13, (11)] was assessed during pregnancy and at 6 months postpartum (and between both time measurements for non-parents), with a positive difference of >2 points between the measures being the criterion of exclusion (i.e., this criterion corresponds to the cut-off for assessing a significant increase in depression). This scale has the advantage of being applied to both parents and non-parents and has been used successfully in perinatal research (12). No participants were excluded based on this criterion. At the last wave of data collection, (non-)parents were asked to select life events that had emotionally affected them in the last year (a relative's death, marital conflicts, breakup of a romantic relationship, loss of a job, and diagnosis of a serious illness in a close relative or in oneself) and assess the emotional impact of each event (13, 14) on a 5-point scale (1 = *not at all affected* and 5 = *extremely affected*). The criteria for exclusion we used was the following: a mean of at least three points across all five events, which was the cut-off to conclude to a significant emotional impact of the experienced events. Three parental couples were excluded based on this criterion.

### Analytical Strategy

To examine the developmental course of parental couples' EI during childbirth, we used the Actor-Partner Interdependence Model [APIM; (15, 16)], a data analytic approach designed to deal with dyadic data through repeated measures. Since our study focused on the development of parental couples rather than the comparison between mothers and fathers, the dyad members were considered to be indistinguishable and, consequently, the terms “actor” and “partner” are used in the results section to refer to both members of the couple. A two-level hierarchical linear modeling [HLM 7.00; (17)] was used: The level 2 data referred to couple variables while the level 1 data referred to all variables that did not include couple information. Three types of predictor variables were included: between-dyads variables, within-dyads variables, and mixed variables (16). A between-dyads variable is one for which scores were the same for both members of the couple but differed from couple to couple (primiparity and multiparity). In contrast, a within-dyads variable is a difference within the couple but a similarity between couples (gender). A mixed predictor variable corresponds to variation both within the couple and between couples, indexed here by the partner's EI development and age (i.e., a control variable). The partner's EI development was introduced as a time-varying covariate in the model predicting the actor's EI. Each time-varying covariate had two sources of variation; therefore, it was treated as two variables instead of one (18). These two sources of variation were likely to have differential effects on the outcome: a between-person effect and a within-person effect, respectively. The time-varying covariate was within-person centered in order to address bias due to unobserved heterogeneity or unmeasured factors that varied across individuals and had a consistent effect over time on the construct of interest (19). The between-person effect concerned the effect on EI of stable individual differences between partners (20). To obtain the between-partner effect, the average level of each partner's EI scores over the three assessment waves was calculated and added as a predictor. This procedure was used to examine the pure effect of change in the time-varying covariate over time (as its mean level was controlled for). In short, the following analyses were conducted: (a) analyses of the missing data, (b) preliminary analyses, (c) APIM analyses, and (d) comparison between the developmental trajectories of parents and non-parents.

## Results

### Missing Data

No attrition occurred between T1 (i.e., pregnancy) and T2 (i.e., 6 months postpartum), yet there was attrition of 64 parents (15.8% of the sample) between T2 and T3 (i.e., 1 year postpartum). Because attrition is common in longitudinal studies, HLM estimates were based on all the available data with the assumption that the missing data were random (21). Statistical comparisons between parents who dropped out and parents who completed the three waves revealed no systematic significant differences in the between-dyads variable (parental group) under investigation [$\chi_{\left(1,\;404\right)}^{2}$ = 0.03, *p* = 0.87] but significant differences between women and men [$\chi_{\left(1,\;404\right)}^{2}$ = 4.18, *p* = 0.04] with a slightly higher tendency to drop out for men.

### Preliminary Analyses

The means and standard deviations of the outcome variables and the Pearson correlation coefficients examining the stability of the repeated measures over time are presented in Tables 1, 2.

**Table 1:** Descriptive statistics—emotional intelligence variables.

	**Global EI**			**Self-control**			**Well-being**			**Emotionality**			**Sociability**		
	**Mean (*****sd*****)**			**Mean (*****sd*****)**			**Mean (*****sd*****)**			**Mean (*****sd*****)**			**Mean (*****sd*****)**		
	**T1**	**T2**	**T3**	**T1**	**T2**	**T3**	**T1**	**T2**	**T3**	**T1**	**T2**	**T3**	**T1**	**T2**	**T3**
Parents	3.65 (0.41)	3.66 (0.45)	3.66 (0.47)	3.41 (0.51)	3.44 (0.52)	3.45 (0.55)	3.92 (0.58)	3.90 (0.62)	3.88 (0.63)	3.73 (0.55)	3.73 (0.63)	3.73 (0.63)	3.49 (0.58)	3.49 (0.57)	3.50 (0.58)
Mothers	3.63 (0.40)	3.68 (0.46)	3.65 (0.48)	3.26 (0.48)	3.37 (0.53)	3.33 (0.55)	3.89 (0.57)	3.88 (0.65)	3.85 (0.65)	3.84 (0.54)	3.86 (0.63)	3.82 (0.67)	3.48 (0.57)	3.49 (0.60)	3.50 (0.60)
Fathers	3.68 (0.43)	3.64 (0.43)	3.67 (0.45)	3.55 (0.48)	3.52 (0.50)	3.58 (0.53)	3.96 (0.59)	3.92 (0.57)	3.90 (0.60)	3.62 (0.54)	3.59 (0.61)	3.63 (0.56)	3.50 (0.59)	3.48 (0.54)	3.50 (0.55)
Primiparous	3.67 (0.42)	3.68 (0.44)	3.67 (0.47)	3.40 (0.52)	3.44 (0.54)	3.45 (0.57)	3.95 (0.57)	3.92 (0.59)	3.88 (0.63)	3.75 (0.57)	3.76 (0.64)	3.75 (0.65)	3.51 (0.58)	3.52 (0.57)	3.53 (0.58)
Multiparous	3.62 (0.40)	3.62 (0.46)	3.63 (0.46)	3.43 (0.46)	3.45 (0.48)	3.47 (0.51)	3.86 (0.60)	3.84 (0.66)	3.85 (0.61)	3.67 (0.49)	3.65 (0.62)	3.69 (0.58)	3.44 (0.57)	3.42 (0.58)	3.44 (0.56)
Nonparents	3.57 (0.37)	3.56 (0.39)		3.26 (0.53)	3.26 (0.49)		3.82 (0.50)	3.78 (0.56)		3.68 (0.50)	3.69 (0.51)		3.45 (0.54)	3.47 (0.54)	
Childl. wom.	3.52 (0.38)	3.53 (0.41)		3.12 (0.52)	3.16 (0.49)		3.79 (0.52)	3.76 (0.60)		3.73 (0.51)	3.74 (0.54)		3.37 (0.55)	3.39 (0.52)	
Childl. men	3.65 (0.35)	3.61 (0.36)		3.48 (0.49)	3.40 (0.46)		3.87 (0.46)	3.81 (0.49)		3.59 (0.46)	3.62 (0.45)		3.56 (0.52)	3.57 (0.54)	

*EI, Emotional Intelligence; n = 402 parents (201 mothers and 201 fathers); n = 215 non-parents (125 women and 90 men). Child. wom., Childless women; Child. men, Childless men*.

**Table 2:** Parents and non-parents' Pearson Correlation Coefficients examining the stability of the repeated measures over time.

	**Global EI**		**Self-control**		**Well-being**		**Emotionality**		**Sociability**	
	**T1**	**T2**	**T1**	**T2**	**T1**	**T2**	**T1**	**T2**	**T1**	**T2**
**Parents**										
T2	0.65^***^	-	0.64^***^	-	0.70^***^	-	0.69^***^	-	0.68^***^	-
T3	0.59^***^	0.66^***^	0.62^***^	0.63^***^	0.62^***^	0.67^***^	0.60^***^	0.68^***^	0.62^***^	0.67^***^
**Non-parents**										
T2	0.76^***^	-	0.78^***^	-	0.72^***^	-	0.63^***^	-	0.81^***^	

*EI, Emotional Intelligence*.

*** *p < 0.001. Child. wom., Childless women; Child. men, Childless men*.

### APIM Results

The APIM results for parents are presented in Table 3: (a) the intraindividual development of EI over time (i.e., slope value), (b) the influence of the parental group (i.e., primiparity and multiparity) as a between-dyads variable, and (c) the association between the actor's EI development and that of his or her partner. For the purpose of the study, the time variable was expressed in the metric of months. The exact difference of time between waves for each participant was respected, making it possible to observe any changes in EI between these three waves of measurement.

**Table 3:** APIM results: coefficients of the intercepts, linear changes (slopes), and predictors of parents' EI change, i.e., (a) between-dyads variable (parental group), (b) within-dyads variable (gender), and (c) mixed variables (partner's level of EI and age).

	**Global EI**			**Self-control**			**Well-being**			**Emotionality**			**Sociability**		
	**Coeff**.	**SE**	***t* (1, 662)**	**Coeff**.	**SE**	***t* (1, 662)**	**Coeff**.	**SE**	***t* (1, 662)**	**Coeff**.	**SE**	***t* (1, 662)**	**Coeff**.	**SE**	***t* (1, 662)**
Intercept	3.67^**^	0.04	85.02	3.44^**^	0.04	78.37	3.91^**^	0.06	65.08	3.74^**^	0.05	68.43	3.51^**^	0.05	64.56
Slope	0.00	0.00	0.72	0.01^*^	0.00	2.31	−0.00	0.00	−1.06	0.00	0.00	0.21	0.00	0.00	1.11
Between–dyads var.															
Parental group	−0.00	0.00	−0.24	−0.00	0.00	−0.20	−0.00	0.00	−1.63	0.00	0.00	0.20	0.00	0.00	0.52
Within–dyads var.															
Gender	0.01	0.01	0.82	−0.00	0.01	−0.57	−0.00	0.01	−0.74	0.02^*^	0.01	2.37	0.01	0.01	0.78
Mixed variables															
Partner's level															
Within	−0.26^**^	0.03	−8.03	−0.23^**^	0.03	−6.90	−0.20^**^	0.03	−5.92	−0.38^**^	0.03	−12.04	−0.25^**^	0.03	−7.41
Between	−0.62^**^	0.04	−15.71	−0.71^**^	0.04	−17.55	−0.68^**^	0.04	−16.86	−0.54^**^	0.04	−14.25	−0.66^**^	0.04	−16.13
Age	0.00	0.00	0.50	0.00	0.00	1.09	−0.00	0.00	−0.85	0.00	0.00	0.42	0.00	0.00	1.03
Deviance	533.03			910.27			1171.71			1078.95			1068.92		

*EI, Emotional Intelligence; Parental group as coded in −1 = multiparous and 1 = primiparous parents; Gender as coded in −1 = father and 1 = mother*.

*N = 408*.

* *p < 0.05*,

** *p < 0.001. Child. wom., Childless women; Child. men, Childless men*.

**Figure 1:**
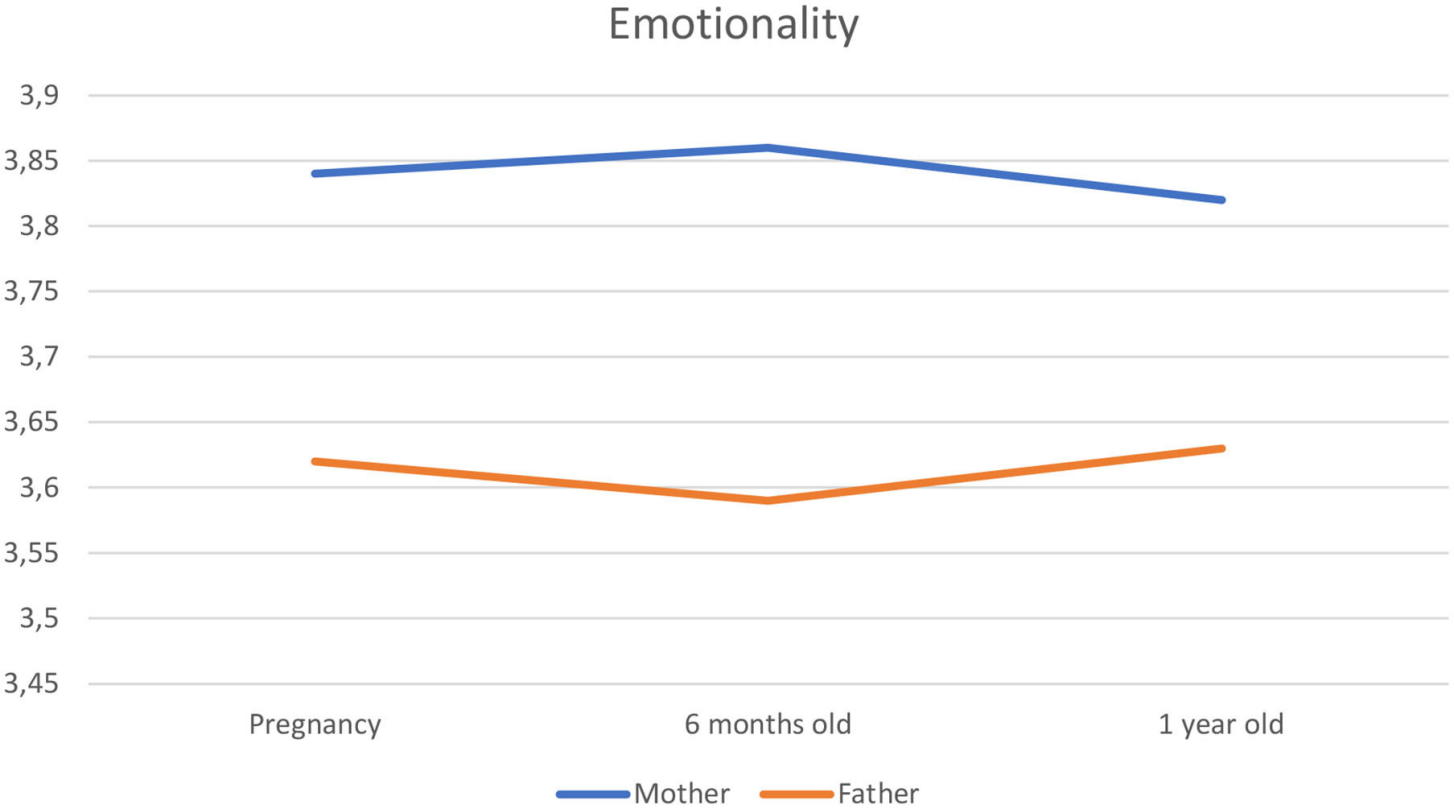
Actor—partner interdependence for emotionality across childbirth.

First, non-significant slope values indicated that almost all EI factors remained stable over time around childbirth, except for Self-Control that slightly increased (β = 0.01, *SE* = 0.00, *t* = 2.31, *p* = 0.02). Second, the results did not show any effect of the parental group on EI factors: Primiparous and multiparous parents followed the same developmental trajectory around childbirth. Third, the results showed an effect of 0.02 (*SE* = 0.01, *t* = 2.37, *p* = 0.02) of gender for Emotionality: Mothers' Emotionality tended to slightly increase while fathers' Emotionality tended to slightly decrease around childbirth. Moreover, F-test showed a significant difference at the baseline level between mothers and fathers for Self-Control [*F*_(1, 403)_ = 36.02, *p* < 0.001] (i.e., fathers displaying greater scores than mothers) and Emotionality [*F*_(1, 403)_ = 17.04, *p* < 0.001] (i.e., mothers displaying greater scores than fathers). No significant difference was found at the baseline for global EI [*F*_(1, 403)_ = 1.00, *p* = 0.32], Well-Being [*F*_(1, 403)_ = 1.42, *p* = 0.23], and Sociability [*F*_(1, 403)_ = 0.17, *p* = 0.68]. Finally, there was a negative association between EI development of the actor in a parental couple and his or her partner's EI development. For every unit of change in their partner's level (i.e., every unit of deviation from the person-specific mean) per month, there was a contrary change in the actor's global EI (β = −0.26, *SE* = 0.03, *t* = −8.03, *p* < 0.001), self-control (β = −0.23, *SE* =0.03, *t* = −6.90, *p* < 0.001), well-being (β = −0.20, *SE* = 0.03, *t* = −5.92, *p* < 0.001), emotionality (β = −0.38, *SE* = 0.03, *t* = −12.04, *p* < 0.001), and sociability (β = −0.25, *SE* =0.03, *t* = −7.41, *p* < 0.001). Figure 1 illustrates this actor-partner interdependence for Emotionality across childbirth.

We also analyzed the developmental trajectory of EI components for non-parental couples. Because only two waves of data were collected for non-parents, it was impossible to use HLM and we used a repeated measure ANOVA. As expected, non-parents did not show any EI development between Time 1 and Time 2 [*F*_(1, 213)_ = 0.51, *p*= 0.48; *F*_(1, 213)_ = 0.03, *p* = 0.87; *F*_(1, 213)_ = 2.64, *p*= 0.11; *F*_(1, 213)_ = 0.12, *p*= 0.73; *F*_(1, 213)_ = 0.63, *p*= 0.43, for global EI, self-control, well-being, emotionality, and sociability, respectively]. When their partners' attachment development was included as a covariate of intraindividual change in the model, there was still no apparent effect [*F*_(1, 62)_ = 0.85, *p* = 0.36; *F*_(1, 62)_ = 4.10, *p* = 0.05; *F*_(1, 62)_ = 0.14, *p* = 0.71; *F*_(1, 62)_ = 0.15, *p* = 0.71; *F*_(1, 62)_ = 0.10, *p* = 0.93, for global EI, self-control, well-being, emotionality, and sociability, respectively].

### Comparison Between the Developmental Trajectories of Parents and Non-parents

To compare the developmental trajectories of parents and non-parents, we (a) analyzed a potential selection effect by comparing differences between both groups at baseline (i.e., Time 1) and (b) compared the two developmental trajectories. First, *F*-test showed a significant difference on the baseline between parents and non-parents for global EI [*F*_(1, 619)_ = 6.42, *p* = 0.01], self-control [*F*_(1, 619)_ = 11.20, *p* < 0.001], and well-being [*F*_(1, 619)_ = 4.57, *p* = 0.03], with parents displaying greater scores than non-parents. No significant difference on the baseline between parents and non-parents was found for Emotionality [*F*_(1, 619)_ = 1.45, *p* = 0.23] and Sociability [*F*_(1, 619)_ = 0.79, *p* = 0.38]. We also compared differences on the baseline between primiparous parents, multiparous parents, and non-parents. F-test showed a significant difference on the baseline between primiparous parents and non-parents for global EI [*F*_(1, 619)_ = 3.79, *p* = 0.02] and well-being [*F*_(1, 619)_ = 3.43, *p* = 0.03], with primiparous parents displaying greater scores than non-parents. Later, the developmental trajectories of parents and non-parents were compared using the two (first) waves of data with a repeated measures design. No difference appeared between parents' and non-parents' trajectories for well-being [*F*_(1, 616)_ = 0.26, *p* = 0.61], emotionality [*F*_(1, 616)_ = 0.08, *p* = 0.78], and sociability [*F*_(1, 616)_ = 0.31, *p* = 0.58]. However, F-test showed significant differences for global EI [*F*_(1, 616)_ = 8.23, *p* < 0.001] and self-control [*F*_(1, 616)_ = 17.08, *p* < 0.001], with slight increases for parents over time.

## Discussion

The main purpose of the current study was to examine the developmental changes of parents' trait EI from before childbirth to 6 months and 1 year following childbirth, compare whether this change was similar for primiparous vs. multiparous parents, and finally, compare potential change in EI with that of non-parents. Across all tests, we controlled for the partner's change in EI.

### Overall Stability of EI Around Childbirth, Except for Increase in Self-Control

Childbirth may be one of the most challenging life events couples face. In this study, however, parents did not show dramatic changes in EI after childbirth. The parents' growth curve was, on average, flat. The coefficient of the intraindividual change was near 0.00. Moreover, parents' and nonparents' EI trajectories did not differ from each other: The two groups showed no significant difference in Well-Being, Emotionality, or Sociability.

Therefore, our first hypothesis regarding an increase of parents' EI after childbirth was not supported by the study results, with the exception being an increase in Self-Control. Due to the descriptive nature of our paper, we cannot explain the exact processes underlying the stability of almost all EI factors.

However, we can propose an explanation tapping into anticipatory changes, i.e., the possibility that EI changes occurred before childbirth (22). Indeed, parents could have anticipated the event: They had at least nine months to prepare for childbirth and even longer if the event was planned, which would explain the absence of EI change in our study. As such, new social roles (i.e., as understood by the SIP) could have been developing already during pregnancy. For example, parents may have already started to demonstrate greater emotional stability for the sake of a calm pregnancy.

Although a majority of EI factors remained stable around childbirth, our results showed an increase in Self-Control after childbirth, whereas no such changes were observed among non-parents. Previous research has already demonstrated the stressful nature of childbirth (23), as evidenced by increasing cortisol levels, housework, family imbalance, and anxiety for the baby's development and heath. The child's crying and screaming, the lack of sleep, and all life changes consecutive to childbirth constantly strain parents' regulatory abilities, which would improve accordingly. This improved Self-Control would then enable better emotion and stress management, which would allow new parents to better adapt to the new family structure and take care of the baby's needs. In addition, this finding resonates with the SIP (8), which states that the investment in parenthood may lead to increasing family expectations. Such expectations would lead to an increase in self-control which may be considered a form of emotional growth.

### Same Developmental Trajectory for Primiparous and Multiparous Parents

Our results showed that primiparous and multiparous parents followed the same developmental trajectory around childbirth and had the same EI score at the baseline level. This result led to two explanations. First, we may suppose an increase in Self-Control only during the first years after childbirth, followed by a return to the baseline. Roberts et al. (24) showed that an exposure to specific contingencies may cause change in traits. As such, childbirth leads temporarily to a more difficult environment (e.g., baby's crying, breastfeeding), which could lead to transient SC changes. Second, both the Transition to Parenthood Hypothesis and New Baby Hypothesis were disconfirmed: both events do not lead to dramatic changes in EI.

### Weak Within-Couples Changes

Our results showed that mothers and fathers tend to follow the same developmental trajectory around childbirth, except for Emotionality. Concerning Emotionality, when comparing mothers' and fathers' scores at the baseline level, we found mothers to have higher levels of Emotionality than fathers. This result echoes the Western norms which consider the free expression of emotion to be “unmanly” (25). Moreover, our results indicated significant differences between mothers' and fathers' Emotionality developmental trajectory: Mothers' Emotionality increased while fathers' Emotionality decreased around childbirth. Consequently, childbirth may lead to further polarization of emotion perception and expression between the two sexes.

### Existence of Between-Couples Changes

The Between-Couples Changes Hypothesis was supported by the study results: Both partners showed the same developmental trajectory but this trajectory differed from couple to couple. The childbirth experience cannot be understood outside the couple. There was a significant negative association between the actor's and the partner's development around childbirth, whereas no such association was found for non-parents. Childbirth pushes parents to function in dyad rather than individually. Compensatory effects may be observed between both parents in terms of emotional management of parenting: When one partner cannot cope emotionally with parenting, the other partner would compensate and better manage the emotional aspects of parenting. On the other hand, having an emotionally competent partner may potentially slow down one's own emotional development.

### Perinatal Mental Health, Limitations, and Research Highlights

The perinatal mental health literature highlights the association between maternal mood instability and emotion dysregulation during the perinatal period, parenting stress, and dysfunctional mother-baby interactions [e.g., (26)]. As a consequence, many studies have focused on the mother's mental health without considering within- and between-couples dynamics. Our study showed compensation effects in EI development between the mother and the father, suggesting potential new avenues to refine current models in perinatal mental health. Now, this dyadic compensatory effect should also be studied in a long-term perspective in order to be able to observe potential risks of maintaining rigid positions in this dyadic process (i.e., one partner always managing one's emotions while the other one not).

The first limitation of this study is related to mental representations during pregnancy and anticipatory changes. Before childbirth, parental couples tend to plan for and imagine their future child (27). Therefore, we may suppose that EI change could also appear during pregnancy. Ideally, we should follow non-parents until the point where they become parents. A second limitation is the absence of certain predictors of intraindividual change in EI. It would be interesting to include maternity and paternity leave as a predictor. International collaborative studies should be conducted since the length of parental leave differs across countries. Finally, from a developmental point of view, it would be interesting to conduct a larger life-span study which would include short-term (i.e., intensive longitudinal study) as well as long-term data, and the same number of waves of data collection between parents and nonparents. This would allow us to observe (a) non-linear EI development, (b) temporary variations of EI, and (c) the potential reversibility of change [e.g., (22)]. Although preliminary and awaiting replication and extension, this study provides the first data to investigate parental EI development around childbirth on a large sample size and with the presence of a control group.

## Data Availability Statement

The raw data supporting the conclusions of this article will be made available by the authors, without undue reservation.

## Ethics Statement

The studies involving human participants were reviewed and approved by University of Louvain. The patients/participants provided their written informed consent to participate in this study.

## Author Contributions

SG collected the data and wrote the first draft of the paper. JG, MM, and PV made many modifications relative to the field of competence (Trait EI for MM and PV and perinatality for JG). All authors contributed to the article and approved the submitted version.

## Conflict of Interest

The authors declare that the research was conducted in the absence of any commercial or financial relationships that could be construed as a potential conflict of interest.

## Appendix

**Table:**

Factorial, subscales structure, and examples of items of the TEIQue (1).		
**Factor facets**	**High scorers perceive themselves as…**	**Examples of items**
**Well-being**		
Self-esteem	Successful and self-confident	I feel that I have a number of good qualities.
Trait happiness	Cheerful and satisfied with their lives	I generally don't find life enjoyable.
Trait optimism	Confident and likely to “look on the bright side” of life	I generally believe that things will work out fine in my life.
**Self-control**		
Emotion regulation	Capable of controlling their emotions	I usually find it difficult to regulate my emotions.
Stress management	Capable of withstanding pressure and regulating stress	On the whole, I'm able to deal with stress.
Low impulsiveness	Reflective and less likely to give in to their urges	I consider all the pros and cons before making a decision.
**Emotionality**		
Emotion perception	Clear about their own and other people's feelings	Many times, I can't figure out what emotion I'm feeling.
Emotion expression	Capable of communicating their feelings to others	Expressing my emotions with words is not a problem for me.
Relationship skills	Capable of having fulfilling personal relationships	Those close to me often complain that I don't treat them right.
Empathy	Capable of taking someone else's perspective	I often find it difficult to see things from another person's viewpoint.
**Sociability**		
Social competence	Accomplished networkers with excellent social skills	I generally find it difficult to express myself clearly.
Emotion management	Capable of influencing other people's feelings	I'm usually able to influence the way other people feel.
Assertiveness	Forthright, frank, and willing to stand up for their rights	I often find it difficult to stand up for my rights.
